# Effective spot size parameters for Acuros dose calculation algorithm using enhanced leaf modelling: Estimation based on small rectangular MLC fields

**DOI:** 10.1002/acm2.70315

**Published:** 2025-11-07

**Authors:** Antonella Fogliata, Antonella Stravato, Marco Pelizzoli, Francesco La Fauci, Pasqualina Gallo, Andrea Bresolin, Luca Cozzi, Giacomo Reggiori

**Affiliations:** ^1^ Radiotherapy and Radiosurgery Department Humanitas Research Hospital IRCCS Milan‐Rozzano Italy; ^2^ Medical Physics Department A.O. San Giovanni‐Addolorata Roma Italy; ^3^ UniCamillus International Medical University in Rome Roma Italy

**Keywords:** Acuros, effective spot size, output factors, rectangular small fields, small fields

## Abstract

**Background and Purpose:**

Small field dosimetry plays a critical role in stereotactic radiotherapy, particularly for elongated fields shaped by multileaf collimators (MLCs). This study aims to optimize the Effective Spot Size (ESS) parameters in the Acuros dose calculation algorithm (Eclipse v18.0, with the enhanced leaf modelling ELM for MLC modelling) for accurate dose calculations of small, rectangular MLC‐shaped fields. Measurements were based on the TRS‐483 protocol and a recently proposed equivalent square field size determination method.

**Methods:**

Field output factors (FOFs) were measured using a Varian TrueBeam linear accelerator with HD‐MLC for 6 MV and 10 MV beams (both flattened and unflattened). Symmetric square and rectangular fields (0.5–4 cm sides) were shaped by the MLC, with jaws fixed at 4.4 × 4.4 cm^2^ to minimize scatter variation. Measurements were performed at isocenter, 10 cm depth using three detectors: microDiamond, PinPoint3D, and DiodeE. The microDiamond detector results were used for ESS optimization due to its complete output correction factor availability. FOFs were calculated in Eclipse v18.0 using Acuros with varying ESS values (0–1.5 mm). The optimal ESS was determined by minimizing FOF differences between calculated and measured values for extreme elongated field sizes in the studied range (0.5 × 4 cm^2^ and 4 × 0.5 cm^2^).

**Results:**

The optimized ESS parameters minimized FOF discrepancies between calculated and measured doses for elongated fields, improving calculation accuracy compared to vendor‐recommended settings (ESSx = 0.5 mm, ESSy = 0.7 mm). Profiles and penumbra analyses supported these findings.

**Conclusions:**

ELM in Eclipse v.18 improves MLC modelling by representing rounded leaf ends. Only ESS parameters remain user‐tunable. A method to determine the ESS for the Acuros algorithm is presented, resulting in accurate algorithm configuration for elongated small fields shaped by an HD‐MLC. Results are presented using 6 MV and 10 MV beams, both flattened and unflattened for an HD‐MLC Varian. ESS affects output factors and penumbra, with dose calculations matching measurements within 0.2% ± 0.4% and a maximum difference of 1.1%.

## 1 INTRODUCTION

Small field measurements are crucial for commissioning and verifying treatment planning systems (TPS), especially for stereotactic radiotherapy, where very small target volumes are treated. The precision of small field dosimetry significantly impacts the accuracy of calculated dose and monitor units (MU). Measurement in small fields remains a complex task due to its physics (lack of lateral charge particle equilibrium, partial occlusion of the radiation source, detector relative to field size). In 2008, a formalism was published,^1^ and in 2014, an IAEA code of practice was made available, the TRS‐483,^2^ where a chapter is dedicated to the output factor measurements in small fields. The recommendations are based on the application of an output correction factor (OCF) to the detector readings ratio, which depends on the detector type and the equivalent square field size obtained from the dosimetric field size. The latter is described in the TRS‐483 with the equivalent area method,^2^, ^3^ which works only when the ratio of the field sides is in the range of (0.7,1.4). For elongated fields, the Sterling formula (four times the field area divided by its perimeter)^4^, ^5^ re‐proposed by Das et al.^6^, ^7^ for small fields improves the estimation of the equivalent square field, leaving, however, discrepancies for fields smaller than 1 cm. Ringholz^8^ estimated the equivalent square field size by optimizing the parameters of a kernel accounting for the primary and scattered radiation. Recently, the proposal of an empirical formula^9^ by modifying the Sterling approach led to results compatible with the Ringholz estimations based on physical interactions. The proposed formula is also consistent with Bjängard's suggestion^10^ of applying a correction factor to the Sterling formula, which depends on the ratio of the two field sides for small fields. This approach allows for accurate measurement of output factors for small fields, also in the conditions of small elongated field settings.

While some studies analyze small square fields following the formalism (comprehensive bibliography can be found in the Code of Practice), there is currently a lack of research on elongated fields, mainly due to the large uncertainty in output determination based on the TRS‐483 protocol for these specific cases.

Similarly, a high level of accuracy is required in dose calculations performed by the TPS. In the common clinical practice, the treatments are delivered with different techniques, mostly intensity modulation with fixed beams, IMRT, or rotational arcs, VMAT, based on the MLC setting. Therefore, the accuracy of dose calculation algorithms must be carefully evaluated for MLC‐shaped beams, taking into account that different collimating devices contribute differently to scattered radiation. Current TPSs are based on fluence modelling,^11^ which ideally traces the beam rays from the source through the MLC leaf ends to ensure high accuracy. In most cases, approximations follow the LoSasso approach,^12^, ^13^ where the beam fluence is modified by virtually shifting the leaf tip positions, on each side of the field, by half of the dosimetric leaf gap (DLG), which is dosimetrically determined from opposed closed leaves. However, this modelling approach may be insufficient, especially when dealing with very small collimated fields, where the issue of partial occlusion of the radiation source becomes significant.^2^, ^14^ Such modelling was implemented in the Eclipse TPS (Varian Medical Systems) up to version 17, where the dosimetric leaf gap (DLG) value was measured according to a vendor‐defined procedure and input manually by the user. More recently, starting from version 18.0, Eclipse introduced the enhanced leaf modelling (ELM), which revolutionizes the LoSasso approach by explicitly modelling the detailed leaf geometry of Varian MLCs: the Millennium, HD‐MLC and the dual‐layer (on Halcyon/ETHOS Linacs) MLCs. The actual MLC leaf shape is accurately represented, ray paths are followed more precisely, the field size is not artificially enlarged, and the user no longer needs to input or fine‐tune the DLG parameter. During the Eclipse algorithm configuration, only two parameters remain for manual input: the Effective Spot Size in *X* and *Y* (ESSx and ESSy). These parameters describe the size of the radiation source and influence the penumbra of the fields generated by the collimated device as well as the output of small fields.^2^, ^14^, ^15^, ^16^ With the implementation of the ELM, the ESS parameters should primarily model the focal spot size, unlike previous approaches, where they also modelled the penumbra. The vendor's suggested ESS values, which have varied across different Eclipse versions, have also been analyzed by several publications.^17^, ^18^, ^19^, ^20^, ^21^

The aim of the current work is to find a procedure to determine the optimal Effective Spot Size parameters for the Acuros dose calculation algorithm, based on measurements of small rectangular MLC‐shaped fields following the TRS‐483 protocol^2^ and the recent method for equivalent square field size determination.^9^

The accuracy of the dose calculation in small elongated fields is then assessed by comparing Acuros version 18.0, configured with the optimal effective spot size parameters, against experimental data.

## 2 METHODS

### 2.1 Linacs and field settings

Small field output factors (FOF) were measured on a Varian TrueBeam linac (Varian Medical Systems, Palo Alto, USA) equipped with an HD‐MLC (2.5 mm leaf width at isocenter), using 6 MV and 10 MV beams in both flattened (X) and flattening filter‐free (FFF) modes. Symmetric square and rectangular fields within a maximum size of 4 × 4 cm^2^ were shaped with the MLC, keeping the jaws set to 4.4 × 4.4 cm^2^. A fixed jaw positioning in all the MLC settings guarantees the same amount of scattered radiation from the jaws back to the monitor chamber. A jaw setting larger than the MLC‐shaped field ensures the MLC is the collimating device. The leaf ends and leaf sides defined the *X* and *Y* directions, respectively, for the YxX fields. The field sizes were set with sides of 0.5, 1, 1.5, 2, 2.5, 3, and 4 cm, for a total of 49 different settings. The fields had the opposed closed leaves positioned under the jaws to eliminate the leakage from closed‐end leaves. The field center was on the beam central axis. All the FOFs were measured at the isocenter, 10 cm depth, for all the available beam qualities.

### 2.2 Detectors

For all fields, three different detectors were used (compliant with the TRS‐483 requirements), mounted on an IBA BluePhantom 2 water phantom (IBA Dosimetry, Schwarzenbruck, Germany), used with its integrated electrometer:

‐ microDiamond type 60019 (PTW, Freiburg, Germany). It is a synthetic single‐crystal diamond detector, with a sensitive volume of 0.004 mm^3^, a radius of 1.1 mm, and 1 micron thick. OCFs are available for fields as small as 0.4 cm side for both 6 and 10 MV beams.^2^
‐ PinPoint3D type 31022 (PTW). It is a cylindrical vented ion chamber, with a sensitive volume of 0.016 cm^3^, a radius of 1.45 mm, and 2.9 mm long. OCFs are provided in the PTW Small Field Dosimetry application Guide^22^ (referring to Casar et al.,^23^ Looe et al.^24^ and Poppinga et al.^25^), and are available for fields as small as 0.8 and 1 cm side for 6 and 10 MV, respectively.
‐ DiodeE type 60017 (PTW). It is an unshielded silicon diode detector, with a sensitive volume of 0.03 mm^3^, an area of 1 mm^2^ and 30 micron thick. OCFs are available for fields as small as 0.5 cm side for 6 MV beams, and as 0.6 cm side for 10 MV.^2^

OCF more than 5% from unity were not reported nor used, according to the TRS‐483 recommendations.

The dosimetric field size, measured as FWHM at the measuring depth, was determined with the microDiamond and used for all the detectors. For each measurement, the beam center was determined via profile acquisition across the main beam axes, and the detector was positioned at the maximum reading of each profile. Output readings were repeated three to five times per point. The accuracy of the microDiamond profile acquisition was checked against film measurements, using Gafchromic EBT3 films.

### 2.3 Output factor, FOF, measurements

The TRS‐483 code of practice^2^ was used to determine the FOF, by correcting the ratio of readings (normalized to 4 × 4 cm^2^ field as for the intermediate field method described in paragraph 3.2.3 of the code of practice), according to the formula^2^:

(1) $$\Omega_{Q_{clin},Q_{msr}}^{f_{clin},f_{msr}}=\frac{M_{Q_{clin}}^{f_{clin}}}{M_{Q_{msr}}^{f_{msr}}}k_{Q_{clin},Q_{msr}}^{f_{clin},f_{msr}}$$

where:

‐ $\Omega_{Q_{clin},Q_{msr}}^{f_{clin},f_{msr}}$ is the FOF of the test (*clinical*) field of size $f_{clin}$ and beam quality $Q_{clin}$, relative to the machine specific reference field $f_{msr}$ (with beam quality $Q_{msr}$), here set to the intermediate 4 × 4 cm^2^ square field;
‐ $\frac{M_{Q_{clin}}^{f_{clin}}}{M_{Q_{msr}}^{f_{msr}}}$ is the ratio of the readings between the test and the *msr* field sizes;
‐ $k_{Q_{clin},Q_{msr}}^{f_{clin},f_{msr}}$ is the output correction factor, OCF, from Tables 26 and 27 of TRS‐483 (or more recent publications), for the specific detector and measured field size.

$f_{clin}$ is the equivalent square field size, determined from the dosimetric field dimensions. In the TRS‐483, it is according to equivalent area ($f_{clin}=\sqrt{A\cdot B}$, where *A* and *B* are the two dosimetric field dimensions). However, since this formula does not hold for elongated fields (out of the range 0.7 < A/B < 1.4), the recent approach for equivalent square field of elongated fields^9^ has been used in this work:

(2) $$\begin{array}{cc} ESF & =\frac{2\cdot min{\left(A,B\right)}^{a}\cdot max{\left(A,B\right)}^{\left(2-a\right)}}{A+B}\\ & =\frac{2\cdot A\cdot B}{A+B}\cdot {\left(\frac{min\left(A,B\right)}{max\left(A,B\right)}\right)}^{a-1} \end{array}$$

with a=1.12 (empirically determined). The *ESF* of Equation (2) has been used as input to determine the OCFs. The TRS‐483 formalism was then applied.

### 2.4 Acuros configurations and calculations

FOFs, as the ratio between the doses of the test and the *msr* (here defined as 4 × 4 cm^2^) MLC‐shaped fields, were computed in the Eclipse treatment planning system version 18.0 (Varian Medical Systems Int., USA) using the Acuros dose calculation algorithm for the same settings as the measurements. A virtual waterphantom (HU = 0, with water material assignment, 1 mm slice spacing to guarantee 1 mm dose calculation resolution also in the longitudinal direction) was prepared. Doses were computed with a calculation grid size of 1 mm (the minimum available).

The Dosimetric Leaf Gap DLG concept has been superseded in version 18.0 of Eclipse by adopting the enhanced leaf modelling (ELM) with the Leaf Gap LG (according to the van Esch notation,^26^ while in Eclipse it is still named DLG). The LG is more related to the mechanical gap between opposed “closed” leaves, and results in a negative value. The DLG in earlier versions was, on the contrary, positive, of the order of a millimeter, and corresponded to the FWHM of the dose peak generated by closed opposed leaves. These completely different concepts make the LG values not comparable with the measured DLG. For details on ELM, refer to the van Esch et al. paper.^26^

The ELM configuration automatically calculates the value of the new LG from a subset of the measurements required for DLG determination: 10 cm depth at isocenter, open and MLC closed fields (for each of the two banks, A and B) with jaws set to 10 × 10 cm^2^; fields with 2, 4, and 20 mm slit openings running along the leaf motion across a 10 × 10 cm^2^ field.

The Effective Spot Size (ESS) parameters in the two collimation directions, ESSx and ESSy, are the only values that require manual input in Acuros version 18. These parameters model the penumbra broadening in each direction by applying a Gaussian smoothing to the energy fluence of primary photons, where ESS corresponds to the width of the Gaussian. ESS influences the absolute dose level (monitor units) for very small fields (≤1 × 1 cm^2^) and affects the calculated penumbra for all field sizes. The ESSx and ESSy values suggested in the Eclipse manual version 18^27^ for Acuros configuration algorithm version 18 of Varian non‐Halcyon unit (i.e. Millennium and HD‐MLC) are:

- ESSx = 0.5 mm and ESSy = 0.7 mm if MLC in field,
- ESSx = 1.0 mm and ESSy = 1.5 mm (with no specification of the collimating system).

These values are also suggested during the configuration, where a dialog box warns the user of a set of parameters different from the vendor's suggestions. Since the patients are clinically treated with the MLC in the beam (the field is never shaped by the jaws), option a) has been initially chosen.

In this study, the beams (6X, 6FFF, 10X, and 10FFF) were configured by varying ESSx and ESSy over a range from 0 to 1.5 mm, with finer increments of 0.1 mm applied between 0.5 and 1.0 mm. The interval was chosen according to the 2016 publication by Fogliata et al.,^19^ and the Eclipse manual version 18.^27^

An identical procedure was followed for all the different Acuros configurations: the same measured datasets were used, and the Eclipse library data for initial conditions were newly loaded at each configuration.

Although both FOF and profiles were analyzed for all ESS settings, the calculated FOF data, being more robust, were used to fine‐tune the ESS parameters.

### 2.5 Output factors and ESS optimization

The FOF were calculated for each ESS setting and compared with the experimental FOF. The variation of the ESSx had the greatest impact on the FOF of the 4 × 0.5 cm^2^ (smallest dimension in the *X* direction), while ESSy variation most affected the 0.5 × 4 cm^2^ field (smallest dimension in the *Y* direction). Therefore, the optimal ESS settings were those that minimized the FOF differences of both 0.5 × 4 cm^2^ and 4 × 0.5 cm^2^. This setting also minimized the range of dose differences, while not necessarily the 0.5 × 0.5 cm^2^ FOF.

Below is the proposed sequence to minimize the number of configurations needed to determine the optimal ESS setting based on FOF:

1. Initial ESS setting, according to the vendor suggestion, option a): ESSx = 0.5 mm, ESSy = 0.7 mm
2. Evaluate the difference between calculated and measured FOF for the two extreme fields, 0.5 × 4 cm^2^ and 4 × 0.5 cm^2^.
3. Vary the ESSx, keeping fixed the ESSy. The ESSx value giving the smallest FOF difference for the 4 × 0.5 cm^2^ field (X = 0.5 cm) is the correct one (ESSx = ESSx’).
4. Vary the ESSy, keeping ESSx = ESSx’. The ESSy value giving the smallest FOF difference for the 0.5 × 4 cm^2^ field (Y = 0.5 cm) is the correct one (ESSy = ESSy’).

### 2.6 Profiles

Profiles in the *X* and *Y* directions across the beam center were exported from Eclipse for all the analyzed fields calculated under each configuration. The dosimetric field size (as FWHM) was compared with the measured data. The penumbra (defined as the distance between the 20% and 80% dose levels in the profile) was also evaluated for both calculations and measurements, with the understanding that the measured penumbra is subject to uncertainty due to detector size and material, while the calculated penumbra may be affected by a relatively coarse calculation grid of 1 mm. These limitations make a fine‐tuning of the ESS parameters based on penumbra matching inaccurate.

### 2.7 Dynamic clinical cases

To bridge to clinical dynamic treatment cases, five patients with brain metastases (one or three, with target diameter ranging from 0.8 to 1.7 cm), for a total of 11 targets, were selected. Single isocenter VMAT plans were optimized for the four analyzed beam qualities, and calculated with the same MUs using configurations with different ESS settings:

- The optimized pair as above.
- ESSx = 0.5 mm and ESSy = 0.7 mm (according to Varian manual version 18, with MLC).
- ESSx = 1.0 mm and ESSy = 1.5 mm (according to Varian manual version 18, with no collimation specification).
- ESSx = 1.5 mm and ESSy = 0.0 mm (according to Varian manual version 16, with MLC).

Box plots will show the differences between settings (b)–(d) relative to setting (a) for the following dosimetric parameters: V_100%_[%], V_98%_[%], V_96%_[%], V_95%_[%] of the PTV (planning target volume).

## 3 RESULTS

### 3.1 Measured field output factors

The final measured FOF used in the following analysis were those acquired with the microDiamond detector. Data are reported in Table 1 for the four beam qualities.

**TABLE 1:** Measured FOF for all the beam qualities.

Energy: 6X								Energy: 6FFF							
X\Y [cm]	0.5	1	1.5	2	2.5	3	4	X\Y [cm]	0.5	1	1.5	2	2.5	3	4
0.5	0.636	0.686	0.700	0.705	0.708	0.710	0.711	0.5	0.662	0.705	0.718	0.723	0.726	0.727	0.729
1	0.737	0.819	0.845	0.857	0.865	0.868	0.872	1	0.757	0.828	0.852	0.863	0.869	0.873	0.877
1.5	0.763	0.858	0.892	0.908	0.918	0.924	0.930	1.5	0.779	0.863	0.894	0.907	0.916	0.922	0.929
2	0.773	0.872	0.912	0.931	0.943	0.951	0.958	2	0.787	0.875	0.910	0.929	0.938	0.946	0.955
2.5	0.777	0.880	0.920	0.945	0.957	0.965	0.975	2.5	0.792	0.882	0.920	0.940	0.953	0.960	0.972
3	0.779	0.883	0.927	0.950	0.965	0.973	0.984	3	0.794	0.887	0.926	0.948	0.962	0.971	0.983
4	0.782	0.888	0.933	0.959	0.975	0.985	1.000	4	0.797	0.893	0.934	0.958	0.973	0.984	1.000

Energy: 10X								Energy: 10FFF							
X\Y [cm]	0.5	1	1.5	2	2.5	3	4	X\Y [cm]	0.5	1	1.5	2	2.5	3	4
0.5	0.534	0.589	0.612	0.623	0.624	0.629	0.629	0.5	0.572	0.626	0.644	0.652	0.655	0.657	0.659
1	0.652	0.748	0.793	0.806	0.817	0.823	0.829	1	0.681	0.773	0.807	0.823	0.831	0.835	0.840
1.5	0.689	0.806	0.858	0.882	0.896	0.904	0.909	1.5	0.715	0.823	0.869	0.890	0.901	0.908	0.914
2	0.701	0.830	0.887	0.919	0.934	0.945	0.952	2	0.727	0.844	0.894	0.920	0.935	0.942	0.951
2.5	0.710	0.840	0.903	0.936	0.952	0.964	0.973	2.5	0.733	0.854	0.909	0.937	0.952	0.962	0.972
3	0.715	0.849	0.910	0.944	0.964	0.975	0.989	3	0.737	0.859	0.916	0.946	0.964	0.973	0.985
4	0.717	0.852	0.919	0.955	0.975	0.989	1.000	4	0.740	0.865	0.924	0.956	0.974	0.987	1.000

**FIGURE 1:**
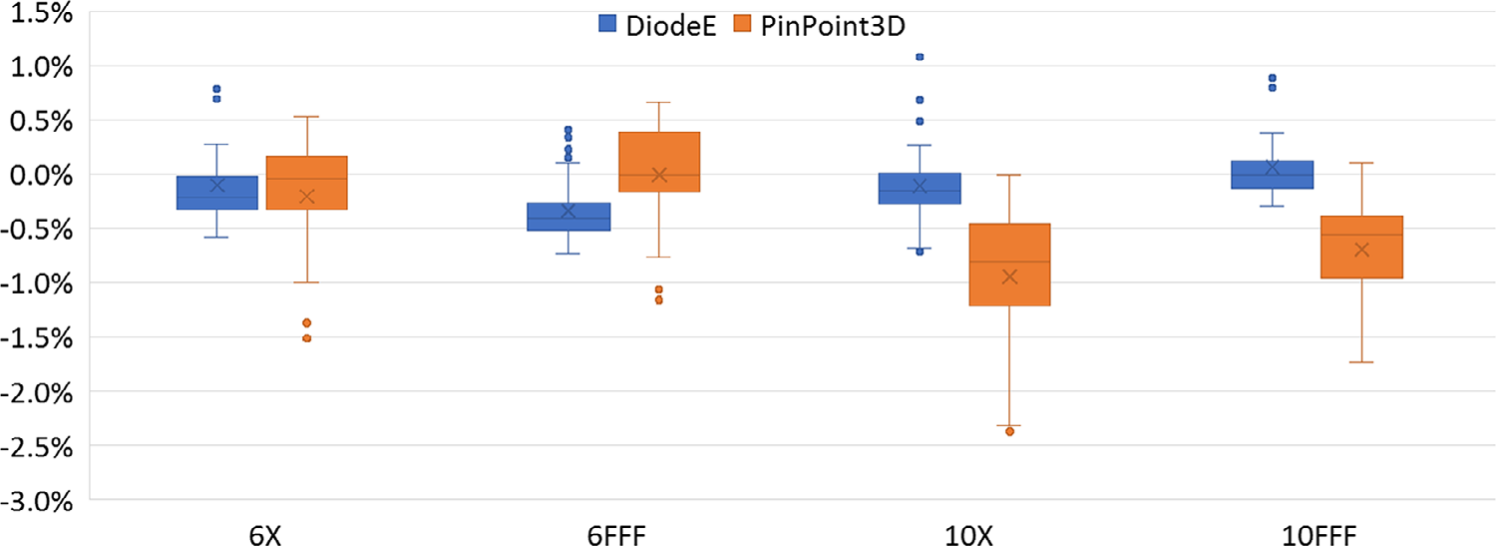
Box plot of the percentage differences between DiodeE and PinPoint3D measured FOF and microDiamond measured FOF. Box plot legenda: the box represents the interquartile range IQR (between first and third quartile); the line inside the box indicates the median; the lines extending from the box are the minimum and maximum values (excluding the outliers); the points out of the lines are the outliers, with values beyond 1.5 times the IQR from the box; the cross indicates the mean value.

Figure 1 reports the box plots of the measured FOF differences between DiodeE/PinPoint3D and the microDiamond measurements, stratified per beam quality, showing a maximum variation of the DiodeE and PinPoint3D relative to the microDiamond FOF of 1.1% and 2.4%, respectively.

The total uncertainty (type A and B combined) was estimated, as 1SD, in 1.8% and 2.4% for the microDiamond and DiodeE, respectively, for the smallest field of 0.5 cm. It decreased to 0.9%, 0.9% and 1.3% for fields from 1 cm field and larger, for the microDiamond, DiodeE and PinPoint3D, respectively.^9^

The selection of the microDiamond FOF measurements for the analysis was primarily based on it being the only detector with OCFs available for all the field sizes and energies used. However, the accuracy of the microDiamond measurements was confirmed by the measurements based on different detectors, with the DiodeE showing similar results (within the uncertainty), and the PinPoint3D presenting a slightly larger spread in the differences.

### 3.2 Leaf gap LG with ELM method

Different ESS values used in the algorithm configuration do not influence the resulting LG, with a maximum variation of only 0.25%. Table 2 presents the LG values calculated during configuration alongside the measured DLG values obtained following the vendor's procedure. The measured DLG parameters are included solely to characterize the MLC calibration of the Linac/MLC used in this study and are not intended for direct comparison, as LG and DLG represent different parameters despite sharing the same name within Eclipse.

**TABLE 2:** Calculated LG (Acuros version 18.0) and measured DLG. Values are given in **mm**. The values are not for comparison.

	6X	6FFF	10X	10FFF
LG from ELM	−0.11	−0.19	−0.09	−0.07
Measured DLG	0.88	0.79	0.92	0.89

### 3.3 Comparison between measured and Acuros calculated FOF

The calculated FOF values depend on the ESSx and ESSy parameters. Table 3 reports the differences between calculated and measured FOF for the 4 × 0.5 cm^2^, 0.5 × 4 cm^2^, and 0.5 × 0.5 cm^2^ fields across different ESS settings for the 6X beam quality (tables for the other beam qualities are reported in Tables S1–S3 of the supplementary material). Based on these results, the optimal ESSx is identified as 0.7 or 0.8 mm (from the 4 × 0.5 cm^2^ field – *X* = 0.5 cm), and the optimal ESSy is 0.7 or 0.8 mm (from the 0.5 × 4 cm^2^ field – *Y* = 0.5 cm). This ESS combination also minimizes the difference for the smallest field (0.5 × 0.5 cm^2^), although this is not always the case. The selected ESS parameters minimize the range of maximum differences across all field sizes, which for the 6X beam range from −0.1% to +0.7%, with an average difference of 0.3% ± 0.2% (1SD).

**TABLE 3:** Percentage differences between calculated and measured FOF as a function of the ESSx and ESSy parameters in mm, for the 4 × 0.5 cm^2^, 0.5 × 4 cm^2^ and 0.5 × 0.5 cm^2^ fields, 6X (Legenda: blue = calculated dose < measured dose, red = calculated dose > measured dose, range for intense blue and red colors: ±5%)

4 x 0.5 cm^2^ (Y = 4 cm, X = 0.5 cm)										0.5 x 4 cm^2^ (Y = 0.5 cm, X = 4 cm)									
ESSy\ESSx	0	0.4	0.5	0.6	0.7	0.8	0.9	1	1.5	ESSy\ESSx	0	0.4	0.5	0.6	0.7	0.8	0.9	1	1.5
0	1.10%				1.11%				1.17%	0	2.60%				−0.03%				−11.29%
0.4			0.75%		0.76%	0.76%	0.77%			0.4			1.42%		−0.03%	−0.03%	−1.90%		
0.5		0.75%	0.75%	0.75%	0.76%	0.76%	0.77%	0.72%		0.5		1.42%	1.42%	1.18%	−0.03%	−0.03%	−1.90%	−1.96%	
0.6			0.60%	0.60%	0.61%	0.61%	0.61%	0.56%		0.6			1.42%	1.18%	−0.03%	−0.03%	−1.90%	−1.96%	
0.7	0.03%		0.03%	0.03%	0.01%	0.01%	0.01%	0.02%	0.07%	0.7	2.65%		1.45%	1.21%	−0.02%	−0.02%	−1.90%	−1.91%	−11.29%
0.8			0.03%	0.03%	0.01%	0.01%	0.01%	0.02%		0.8			1.45%	1.21%	−0.02%	−0.02%	−1.90%	−1.91%	
0.9		−0.83%	−0.83%	−0.83%	−0.84%	−0.84%	−0.84%	−0.84%		0.9		1.45%	1.45%	1.21%	−0.02%	−0.02%	−1.91%	−1.91%	
1			−0.85%	−0.85%	−0.85%	−0.85%	−0.85%	−0.87%	−0.80%	1			1.43%	1.19%	−0.04%	−0.04%	−1.92%	−1.93%	−11.30%
1.5	−6.11%				−6.09%				−1.55%	1.5	2.69%				0.05%				−11.20%
					0.5 x 0.5 cm^2^ (Y = 0.5 cm, *X* = 0.5 cm)									
					ESSy\ESSx	0	0.4	0.5	0.6	0.7	0.8	0.9	1	1.5
					0	4.06%				1.39%				−10.52%
					0.4			2.30%		0.84%	0.84%	−1.09%		
					0.5		2.30%	2.30%	2.07%	0.84%	0.84%	−1.09%	−1.16%	
					0.6			2.51%	1.95%	0.73%	0.73%	−1.20%	−1.27%	
					0.7	3.12%		1.94%	1.70%	0.19%	0.19%	−1.72%	−1.73%	−11.56%
					0.8			1.94%	1.70%	0.19%	0.19%	−1.72%	−1.73%	
					0.9		1.04%	1.04%	0.81%	−0.63%	−0.63%	−2.53%	−2.54%	
					1			1.03%	0.80%	−0.64%	−0.64%	−2.55%	−2.56%	−12.27%
					1.5	−3.58%				−6.01%				−16.94%

Results also show that too small ESS will overestimate the calculated dose, while too large ESS will yield a calculated dose underestimation.

The data exhibited a stepwise pattern when varying the ESS, indicating that identical results were obtained for all fields at ESS values of 0.7 and 0.8 mm, 0.9 and 1.0 mm, 0.4 and 0.5 mm, as well as 0.0 and 0.2 mm. In Table 3, the equivalence of ESS = 0.7 and 0.8 mm is visible.

The estimated optimal ESS settings for all analyzed beam qualities are reported in Table 4, where the 10FFF beam shows a substantial reduction in ESS parameters compared to the other beam qualities.

**TABLE 4:** Optimal ESS parameter estimation for all the beam qualities.

	6X	6FFF	10X	10FFF
ESSx (mm)	0.7–0.8	0.7–0.8	0.6	0.3
ESSy (mm)	0.7–0.8	0.7–0.8	0.7–0.8	0.4–0.5

The differences between calculated (using the optimal ESS) and measured FOF are reported in Table 5 for all field sizes and beam qualities. In summary, Table 6 presents the mean values ± 1 SD (and corresponding ranges) of these differences across all analyzed field sizes for each energy, alongside results obtained using Acuros configurations with vendor‐suggested ESS settings. The vendor's suggestion of the ESS parameters for MLC setting gives, although not optimized, acceptable results.

**TABLE 5:** Difference between calculated and measured FOF for all beam qualities. Acuros is configured with the optimal ESS setting. (Legenda: blue = calculated dose < measured dose, red = calculated dose > measured dose, range for intense blue and red colors: ±2%).

Energy: 6X								Energy: 6FFF							
X\Y [cm]	0.5	1	1.5	2	2.5	3	4	X\Y [cm]	0.5	1	1.5	2	2.5	3	4
0.5	0.2%	0.4%	0.2%	0.1%	0.1%	0.0%	0.0%	0.5	1.0%	1.1%	0.9%	0.7%	0.6%	0.6%	0.5%
1	0.5%	0.7%	0.7%	0.5%	0.2%	0.1%	0.1%	1	0.9%	1.0%	0.9%	0.6%	0.5%	0.3%	0.3%
1.5	0.2%	0.6%	0.5%	0.4%	0.1%	0.0%	−0.1%	1.5	0.7%	0.8%	0.7%	0.6%	0.4%	0.2%	0.2%
2	0.1%	0.6%	0.5%	0.4%	0.2%	0.0%	0.1%	2	0.6%	0.8%	0.8%	0.6%	0.5%	0.3%	0.2%
2.5	0.1%	0.6%	0.6%	0.2%	0.2%	0.1%	0.0%	2.5	0.4%	0.8%	0.7%	0.5%	0.3%	0.3%	0.1%
3	0.0%	0.6%	0.5%	0.4%	0.2%	0.2%	0.2%	3	0.3%	0.7%	0.7%	0.5%	0.3%	0.2%	0.1%
4	0.0%	0.6%	0.6%	0.4%	0.2%	0.1%	0.0%	4	0.2%	0.6%	0.6%	0.4%	0.3%	0.1%	0.0%

Energy: 10X								Energy: 10FFF							
X\Y [cm]	0.5	1	1.5	2	2.5	3	4	X\Y [cm]	0.5	1	1.5	2	2.5	3	4
0.5	0.4%	1.0%	0.3%	−0.2%	0.1%	−0.3%	0.0%	0.5	0.4%	0.3%	0.1%	0.0%	0.0%	−0.1%	−0.1%
1	0.6%	1.3%	0.3%	0.7%	0.3%	0.1%	−0.1%	1	0.0%	−0.2%	−0.3%	−0.4%	−0.4%	−0.4%	−0.5%
1.5	0.2%	0.9%	0.5%	0.3%	0.0%	−0.2%	0.0%	1.5	−0.4%	−0.3%	−0.4%	−0.4%	−0.5%	−0.6%	−0.4%
2	0.5%	0.9%	0.6%	0.0%	0.0%	−0.4%	−0.2%	2	−0.3%	−0.1%	0.0%	−0.1%	−0.3%	−0.3%	−0.2%
2.5	0.2%	1.0%	0.5%	0.2%	0.2%	−0.1%	−0.1%	2.5	−0.3%	−0.1%	−0.1%	−0.1%	−0.1%	−0.1%	−0.1%
3	−0.1%	0.6%	0.6%	0.4%	0.0%	0.0%	−0.3%	3	−0.4%	−0.1%	−0.1%	−0.1%	−0.1%	0.0%	−0.1%
4	0.0%	0.8%	0.5%	0.2%	0.1%	−0.2%	0.0%	4	−0.5%	0.0%	0.0%	0.0%	0.0%	−0.1%	0.0%

**TABLE 6:** Mean ± 1SD, (range) of the difference between calculated and measured FOF over all the analyzed field sizes, square and elongated, for the optimized and vendor‐suggested ESS settings.

Energy	Optimised ESS (Table 4)	Vendor ESS: (0.5/0.7 mm, MLC)	Vendor ESS: (1.0/1.5 mm)
6X	0.3% ± 0.2% [−0.1%, +0.7%]	0.4% ± 0.3% [−0.1%, +1.3%]	−1.7% ± 4.0% [−12.3%, +0.6%]
6FFF	0.5% ± 0.3% [0.1%, +1.1%]	0.5% ± 0.3% [0.0%, +1.1%]	−1.5% ± 4.0% [−12.1%, +0.8%]
10X	0.3% ± 0.4% [−0.4%, +1.3%]	0.3% ± 0.4% [−0.4%, +1.4%]	−1.8% ± 3.6% [−11.9%, +0.6%]
10FFF	0.2% ± 0.2% [−0.6%, +0.4%]	−0.5% ± 0.5% [−1.6%, +0.0%]	−2.5% ± 4.1% [−13.8%, +0.1%]

### 3.4 Comparison between measured and Acuros calculated profiles

The accuracy of the profile is limited on one hand by the dose calculation grid size (minimum 1 mm) and on the other hand by the detector's volume and density during measurements. The measured field size uncertainty is estimated at 0.2 mm as 2SD.^9^ Comparison between profiles acquired with the microDiamond (as used in the current work) and film measurements showed an agreement in the penumbra estimation within the 0.2 mm uncertainty, confirming the suitability of the microDiamond detector for profile measurement, that are possible in the same conditions as output measurement (in waterphantom). An example of the film‐microDiamond profile comparison is shown in Figure S1 of the Supplementary Material. In summary, the comparison between calculation and measurement shall account for such reduced accuracies.

Notwithstanding the ELM modelling, the calculated dosimetric field size and penumbra also depend on the ESS setting.

The measured dosimetric field sizes are larger than the nominal field sizes in the *X* direction (parallel to the leaf motion) and smaller in the *Y* direction (perpendicular to the leaf motion).^9^ The differences between calculated (with optimized ESS) and measured field sizes are presented in Table 7. It is worth noting that a good agreement was observed in the *Y* direction in all the situations, better than that in the *X* direction. The *Y* direction describes the leaf side, driven by the leaf mechanical construction, while the *X* direction describes the leaf end and depends on the MLC calibration and the precise leaf positioning, suggesting machine‐specific characteristics in this direction.

**TABLE 7:** Dosimetric field size differences in mm between calculations (with FOF‐optimized ESS) and measurements, for the 0.5 × 0.5 cm^2^, 1 × 1 cm^2^ and 2 × 2 cm^2^ nominal field sizes.

	6X		6FFF		10X		10FFF	
Field size	*X* (End)	*Y* (Side)	*X* (End)	*Y* (Side)	*X* (End)	*Y* (Side)	*X* (End)	*Y* (Side)
0.5 × 0.5 cm^2^	0.3	0.1	−0.1	0.1	0.3	0.2	0.0	0.0
1 × 1 cm^2^	0.7	0.1	0.5	−0.1	0.5	−0.1	0.4	−0.1
2 × 2 cm^2^	0.3	−0.1	0.2	−0.1	0.1	−0.1	0.3	−0.1

The dosimetric field size increases with ESS, for all fields, but particularly for the smallest 0.5 cm field, where it varied in the *X* direction (for 6X) from 0.59 to 0.68 cm for ESSx from 0 to 1.5 mm, and in the *Y* direction from 0.49 to 0.56 cm for ESSy from 0 to 1.5 mm. The amount of those variations is similar in the two directions, indicating the need for a proper estimation of the source size (the ESS) even for the more stable direction *Y*. Figure S2 of the supplementary material reports the variation of the dosimetric relative to the nominal field size for 0.5 × 0.5, 1 × 1 and 2 × 2 cm^2^, 6X.

**FIGURE 2:**
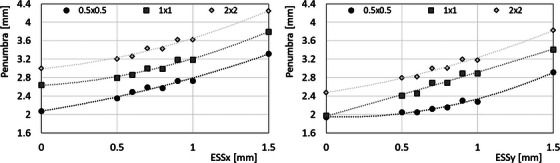
Calculated penumbra as a function of the ESS parameter, *X* direction on the left, *Y* direction on the right, for 0.5 × 0.5, 1 × 1 and 2 × 2 cm^2^ fields from 6X.

Regarding the penumbrae, measured and calculated values (with FOF‐optimized ESS) are reported in Table 8 for the nominal square fields of 0.5 × 0.5, 1 × 1 and 2 × 2 cm^2^. In the worst cases, the difference between calculated and measured penumbrae is 0.2 mm, compatible with the calculation and measurement uncertainties. The penumbra also showed, as expected, increasing values with the ESS, for all field sizes, as shown in Figure 2 for the 6X case.

**TABLE 8:** Mean (left and right) penumbrae in mm: calculations with optimized ESS and measurements with microDiamond, for the 0.5 × 0.5 cm^2^, 1 × 1 cm^2^ and 2 × 2 cm^2^ nominal field sizes.

		6X		6FFF		10X		10FFF	
Field size		X	Y	X	Y	X	Y	X	Y
0.5 × 0.5 cm^2^	Calc	2.6	2.1	2.3	2.1	2.9	2.6	2.5	2.2
	Meas	2.4	2.0	2.2	2.0	2.8	2.6	2.5	2.3
1 × 1 cm^2^	Calc	3.0	2.7	2.6	2.5	3.5	3.2	3.1	2.9
	Meas	2.8	2.5	2.6	2.4	3.5	3.2	3.0	2.8
2 × 2 cm^2^	Calc	3.4	3.0	3.0	2.8	4.1	3.7	3.7	3.4
	Meas	3.3	2.8	3.1	2.6	4.3	3.8	3.8	3.3

### 3.5 Dynamic clinical cases

**FIGURE 3:**
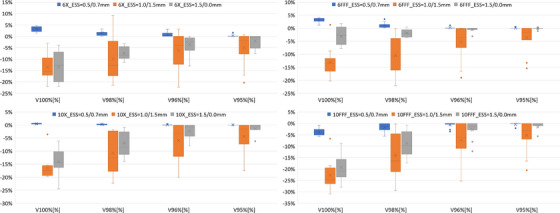
Box plots of the differences between vendor suggested and optimized ESS pairs for some dosimetric parameters of PTV. Box plot legenda: the box represents the interquartile range (IQR) between the first and third quartile; the line inside the box is the median; the lines extending from the box (whiskers) are the minimum and maximum values, excluding outliers (values beyond 1.5 the IQR from the box); the outliers are the data points falling outside the whiskers; the cross indicates the mean value.

The differences of some *V_x_* parameters, between vendor suggested and optimized ESS pairs are presented as box plots in Figure 3. The parameter presenting the largest difference for different ESS is the V_100%_, with differences decreasing with dose level. In general, the values suggested for version 18 and MLC present differences within 5% relative to the optimized ESS values. On the contrary, a systematic underestimation of the volume receiving high dose levels is shown, particularly for ESS values suggested by the vendor in version 18 with no specified collimating device (ESS = 1.0/1.5 mm).

## 4 DISCUSSION

This study evaluated the accuracy of Acuros dose calculations in Eclipse version 18.0 using ELM modelling, focusing on ESS optimization for small square and elongated fields (ranging from 0.5 × 0.5 cm^2^ to 4 × 4 cm^2^) on the beam central axis shaped by an HD‐MLC on a Varian TrueBeam Linac. The beams studied included 6 MV and 10 MV energies, both flattened and unflattened.

The introduction of ELM in Eclipse version 18 significantly improved the MLC modelling by physically representing rounded leaf ends, in place of the previous sharp edge model and DLG shift. The founding testing study from van Esch et al.^26^ proved a very good agreement on calculated profiles compared with film and liquid ion chamber array measurements in static “wallpaper” setting, static narrow MLC strips, and dynamic “zebra crosswalk” setting. The largest improvement of the ELM approach relative to the DLG was shown at large distances (in the x direction) from the beam central axis.

For the HD‐MLC used in the current study, a recent work of Moktan et al.^28^ compared the ELM and DLG models with AAA calculations in the same “zebra crosswalk” as van Esch, confirming the results, and reporting a positive impact of ELM in single‐isocenter multi‐target brain treatments' dose calculation accuracy. Apart from the foundational paper, other publications evaluate the ELM using clinical cases and patient‐specific quality assurance (PSQA), which carries the risk of masking certain effects due to patient anatomy or PSQA system inaccuracies. The differences in the dose calculations for varying ESS in clinical cases presented in this work, although not pointing to the PSQA, which is out of our scope, reported dosimetric important differences in clinical cases. This showed a possible underestimation of the target coverage if an inaccurate ESS pair is used, eventually leading to plan unnecessary larger field sizes, to achieve the visualization of the desired target coverage in the TPS dose distribution.

To date, no systematic study has been published on small static MLC fields (square or elongated) calculated with ELM.

With ELM modeling, the LG is no longer an adjustable parameter, leaving only the ESSx and ESSy parameters manually tunable. Consistent with vendor recommendations, adjusting ESS is critical for achieving agreement between calculated and measured doses in small fields (<1 cm) and penumbral regions.^27^ Extra care must be even taken when selecting detectors and following measurement procedures for both output factors in very small fields and profile measurements, as no detector is ideal.^27^

Different ESS values have been proposed for various algorithm versions. In the DLG‐based implementation of Acuros, there is a notable dependence between ESS and DLG settings, particularly for small fields, as clearly demonstrated by Passal et al.^21^ using a design of experiments methodology. Some publications have investigated the optimal ESS settings for DLG‐based algorithms using small square static beams, also highlighting the interrelationship between DLG and ESS.^17^, ^18^, ^19^, ^21^ Other studies were based on clinical plans and pre‐treatment QA, proposing the ESS and DLG tuning^20^ or checking the implemented setting validity.^21^ Some authors^17^, ^21^ tuned the ESS by matching calculated and measured profiles, notwithstanding the inaccuracies due to the detector volume (and density) effect, probably in consideration of the supposed use of the ESS parameters in the management of the penumbra shape.

The introduction of the ELM needed an update of the studies based on the DLG method. With ELM, the penumbra shape is partially modelled according to the rounded leaf end, potentially leaving ESS adjustment to account primarily for physical spot size effects.

The procedure proposed in this work optimizes the ESS based on accurately measured small elongated fields FOF. Profiles were used as a verification tool, in addition to determining the dosimetric field size required for FOF. The results showed good agreement in field size along the *Y* direction, which is defined by the leaf side. In the *X* direction, the MLC calibration might cause variations in dosimetric field size, so affecting the output and the required ESSx parameter. Moreover, a potential MLC miscalibration (or different calibration) could slightly reduce the agreement between calculations and measurements.

The ESS values suggested by the vendor for version 18 with ELM are close to the optimal ESS settings determined in this study, provided the correct values are used (option a): MLC in the field). However, since these values are not tailored to any specific MLC calibration and status, the correctness of the ESSx parameter should be verified.

Furthermore, the DLG measurements could still be considered an important part of the periodic quality assurance procedure to prevent the use of miscalibrated MLCs.

ELM testing^26^ focused on comparisons between measurements and calculations using a configuration with the ESS set to 0.5 and 0.7 mm, values also in agreement with the work by Sawkey et al.^29^ on measurements of the radiation spot size of TrueBeam linacs. Sawkey et al. results showed, on TrueBeams before 2013, a marked asymmetry in the spot, with sigmas from 0.47 to 1.05 mm. In particular, they found that 6 MV beams (flattened and unflattened) presented an elongated shape, with the major axes 30%–40% larger than the minor axes and oriented 40° from the in‐plane direction. Although the spot size in Eclipse physically represents the focal spot, its implementation causes the spot to rotate along with the collimator to model the collimating device, which does not actually occur in reality. This suggests the importance of a symmetric physical focal spot for accurate calculations even during collimator rotations. Interestingly, in the Sawkey et al. work, the authors describe that the 10FFF spot was 10% smaller than the 10X one. In the current study, the estimated ESS for the 10FFF beam was found to be smaller than the other beam qualities (including 10X), in agreement with their statement.

The ESS adjustment remains critically important in algorithm configurations for Eclipse versions earlier than 18 without ELM. To discuss advances in the more recent version, a comparison of measurements and calculations for Acuros versions 16.1 is summarized in Table 9, which reports the mean, standard deviation, and range of FOF differences across various scenarios: two ESS settings according to the vendor recommendations for version 16.1 in the Eclipse manual of version 16.1 (MLC and not specified, options (a) and (b)), and one ESS setting from manual version 18.0 concerning previous algorithm versions; the last ESS was optimized for Acuros version 16.1, using the measured DLG. Although the procedures and data for the algorithm configurations were identical in all the cases, the differences shown in Table 9 are the results of dose calculations with versions having different modelling, in particular, the DLG/LG (ELM), making such a comparison a comprehensive result between measurement and calculations in the specific algorithm conditions more than a simple evaluation of the ESS.

**TABLE 9:** Mean ± 1SD, [range] of the differences between version 16.1 calculated and measured FOF over all the analyzed field sizes, square and elongated, using vendor‐suggested ESS settings available in different manuals (v. 16.1 and v. 18.0) for version 16.1 algorithms.

Acuros vers. 16.1	6X	6FFF	10X	10FFF
ESS = 1.5,0.0 mm Manual v. 16.1, MLC	0.1% ± 1.9% [−4.3%, +3.2%]	0.6% ± 2.1% [−4.5%, +4.4%]	−0.3% ± 2.0% [−5.1%, +3.0%]	−0.9% ± 2.0% [−6.0%, +1.4%]
ESS = 1.0,1.0 mm Manual v. 16.1	0.3% ± 0.7% [−1.7%, +1.1%]	0.1% ± 0.6% [−1.9%, +0.7%]	−0.3% ± 0.8% [−2.9%, +0.8%]	−1.0% ± 1.2% [−4.6%, +0.0%]
ESS = 0.5,0.7 mm Manual v. 18.0, MLC	0.8% ± 0.6% [+0.1%, +2.6%]	0.5% ± 0.3% [+0.1%, +1.3%]	0.3% ± 0.5% [−0.4%, +1.4%]	−0.4% ± 0.5% [−1.6%, +0.0%]
Optimised (v.16.1)	0.4% ± 0.4% [−0.7%, +1.2%]	0.4% ± 0.3% [−0.3%, +0.9%]	0.2% ± 0.4% [−0.4%, +1.2%]	−0.2% ± 0.2% [−0.6%, +0.3%]
	ESS = 1.1,0.7 mm	ESS = 1.0,0.7 mm	ESS = 0.7,0.7 mm	ESS = 0.5,0.5 mm

The grid size represents an unavoidable limitation for users of the clinical Eclipse system when calculating very small fields. In this work, the minimum grid size of 1 mm in all directions was used. However, for field sizes as small as 0.5 cm per side, this grid resolution is relatively coarse and may theoretically lead to an underestimation of output due to a smoothing (volume averaging) effect.

Limitations of this work are mainly: (i) the optimization of the ESS for the HD‐MLC only, (ii) the missing evaluation of a possible influence of the MLC miscalibration, (iii) the missing evaluation of the same setting at 90 degree collimator, (iv) the fields centered on the central axis only.

Future work will extend this study to other Varian MLCs—Millennium and Dual‐Layer—for which the Enhanced Leaf Modelling (ELM) is available. Additionally, off‐axis fields will be evaluated, as improvements are expected with the ELM implementation.

## 5 CONCLUSION

The introduction of ELM in Eclipse version 18 significantly improves MLC modelling by physically representing rounded leaf ends instead of the previous sharp edge model and DLG shift. The LG parameter in ELM is no longer adjustable, and only the ESS parameters in the *X* and *Y* directions remain tunable by the user.

The study presents a method to determine the ESS parameters for Acuros dose calculations using ELM modelling, enabling accurate algorithm configuration for small square and elongated fields ranging from 0.5 × 0.5 cm^2^ to 4 × 4 cm^2^, shaped by a HD‐MLC on a Varian TrueBeam, leading to a mean difference between calculated and measured doses of 0.2% ± 0.4%. Accurate measurements of FOF for rectangular fields are essential, following the TRS‐483 code of practice, along with a correct estimation of the equivalent square field. The improved accuracy of the algorithm configuration described here may lead to more precise dose calculations in stereotactic radiotherapy planning.

## AUTHOR CONTRIBUTIONS

**Fogliata A., Stravato A., Cozzi L**.: Conceptualization; methodology; supervision; data acquisition and validation; investigation; writing—original draft; writing—review & editing. **Pelizzoli M., La Fauci F., Gallo P., Bresolin A., Reggiori G**.: Data Acquisition and validation; review.

## CONFLICT OF INTEREST STATEMENT

The authors declare no conflicts of interest.

## Supporting information



Supporting Information
