# Supplementary material for: Effective spot size parameters for Acuros dose calculation algorithm using enhanced leaf modelling: Estimation based on small rectangular MLC fields
Source: J Appl Clin Med Phys. 2025 Nov 7;26(11):e70315. doi: 10.1002/acm2.70315 (PMC12593538; doi:10.1002/acm2.70315)

Table S1 – *Percentage differences between calculated and measured FOF as a function of the ESSx and ESSy parameters in mm, for the 4x0.5 cm^2, 0.5x4 cm^2 and 0.5x0.5 cm^2 fields,* ***<u>6FFF</u>****. (Legenda: blue=calculated dose<measured dose, red=calculated dose>measured dose, range for intense blue and red colours: ±5%)*

4x0.5 cm^2 (Y=4 cm, X=0.5 cm)

ESSx \ ESSy	0	0.4	0.5	0.6	0.7	0.8	0.9	1	1.5
0	0.49%				0.50%				0.68%
0.4					0.35%				
0.5			0.35%		0.35%			0.36%	
0.6	0.20%		0.20%	0.20%	0.20%	0.20%	0.20%	0.20%	0.40%
0.7	-0.40%		-0.40%		-0.39%	-0.39%		-0.39%	-0.19%
0.8				-0.40%	-0.39%	-0.39%			
0.9			-1.28%		-1.26%				
1			-1.27%		-1.26%			-1.27%	-1.07%
1.5	-6.11%				-6.10%				-6.08%

0.5x4 cm^2 (Y=0.5 cm, X=4 cm)

ESSx \ ESSy	0	0.4	0.5	0.6	0.7	0.8	0.9	1	1.5
0	3.00%				0.51%				-11.00%
0.4					0.51%				
0.5			1.91%		0.51%			-1.39%	
0.6	3.01%		1.91%	1.68%	0.51%	0.51%	-1.39%	-1.39%	-11.00%
0.7	3.01%		1.89%		0.50%	0.50%		-1.40%	-11.01%
0.8				1.67%	0.50%	0.50%			
0.9			1.89%		0.50%				
1			1.89%		0.50%			-1.41%	-11.01%
1.5	4.12%				1.54%				-10.34%

0.5x0.5 cm^2 (Y=0.5 cm, X=0.5 cm)

ESSx \ ESSy	0	0.4	0.5	0.6	0.7	0.8	0.9	1	1.5
0	3.97%				1.59%				-10.43%
0.4					1.09%				
0.5			2.49%		1.09%			-0.86%	
0.6	3.79%		2.37%	2.16%	0.98%	0.98%	-0.96%	-0.96%	-10.96%
0.7	3.18%		1.84%		0.45%	0.45%		-1.47%	-11.41%
0.8				1.62%	0.45%	0.45%			
0.9			0.97%		-0.39%				
1			0.97%		-0.39%			-2.31%	-12.12%
1.5	-2.57%				-5.05%				-16.35%

Table S2 – *Percentage differences between calculated and measured FOF as a function of the ESSx and ESSy parameters in mm, for the 4x0.5 cm^2, 0.5x4 cm^2 and 0.5x0.5 cm^2 fields,* ***<u>10X</u>****. (Legenda: blue=calculated dose<measured dose, red=calculated dose>measured dose, range for intense blue and red colours: ±5%)*

4x0.5 cm^2 (Y=4 cm, X=0.5 cm)

ESSx \ ESSy	0	0.5	0.6	0.7	0.8	0.9	1	1.5
0	0.73%			0.73%				0.76%
0.3				0.68%				
0.4			0.20%	0.20%	0.20%			
0.5		0.20%	0.20%	0.20%	0.20%		0.20%	
0.6	0.02%	0.03%	0.03%	0.02%	0.02%	0.03%	0.02%	0.04%
0.7			-0.61%	-0.60%	-0.60%			
0.8				-0.60%				
1		-1.48%		-1.51%			-1.51%	-1.48%
1.5	-6.30%			-6.29%				-6.19%

0.5x4 cm^2 (Y=0.5 cm, X=4 cm)

ESSx \ ESSy	0	0.5	0.6	0.7	0.8	0.9	1	1.5
0	2.83%			-0.04%				-10.21%
0.3				-0.04%				
0.4			1.20%	-0.04%	-0.04%			
0.5		1.48%	1.20%	-0.04%	-0.04%		-1.81%	
0.6	2.83%	1.48%	1.20%	-0.04%	-0.04%	-1.80%	-1.81%	-10.21%
0.7			1.20%	-0.04%	-0.04%			
0.8				-0.04%				
1		1.46%		-0.08%			-1.84%	-10.24%
1.5	2.82%			-0.04%				-10.13%

0.5x0.5 cm^2 (Y=0.5 cm, X=0.5 cm)

ESSx \ ESSy	0	0.5	0.6	0.7	0.8	0.9	1	1.5
0	4.14%			1.16%				-9.94%
0.3				0.93%				
0.4			1.82%	0.52%	0.52%			
0.5		2.37%	1.82%	0.52%	0.52%		-1.38%	
0.6	0.02%	0.03%	0.03%	0.02%	0.02%	0.03%	0.02%	0.04%
0.7			1.28%	-0.24%	-0.24%			
0.8				-0.24%				
1		0.62%		-1.13%			-2.96%	-11.93%
1.5	-3.61%			-6.31%				-16.35%

Table S3 – *Percentage differences between calculated and measured FOF as a function of the ESSx and ESSy parameters in mm, for the 4x0.5 cm², 0.5x4 cm² and 0.5x0.5 cm² fields,* ***<u>10FFF</u>****. (Legenda: blue=calculated dose<measured dose, red=calculated dose>measured dose, range for intense blue and red colours: ±5%)*

4x0.5 cm² (Y=4 cm, X=0.5 cm)

ESSx \ ESSy	0	0.2	0.3	0.4	0.5	0.6	0.7	0.8	0.9	1	1.5
0					-0.48%		-0.49%				
0.1					-0.48%		-0.49%				
0.2					-0.48%		-0.49%				
0.3	-0.48%	-0.48%	-0.48%	-0.48%	-0.48%	-0.48%	-0.49%			-0.49%	-0.36%
0.4					-0.93%		-0.94%				
0.5	-0.93%	-0.93%	-0.93%	-0.93%	-0.93%	-0.93%	-0.94%	-0.94%	-0.94%	-0.94%	-0.81%
0.6							-1.09%				
0.7							-1.69%				
0.8							-1.69%				
1							-2.51%				-2.40%
1.5							-7.09%				

0.5x4 cm² (Y=0.5 cm, X=4 cm)

ESSx \ ESSy	0	0.2	0.3	0.4	0.5	0.6	0.7	0.8	0.9	1	1.5
0					-0.13%		-1.58%				
0.1					-0.13%		-1.58%				
0.2					-0.13%		-1.58%				
0.3	1.11%	1.11%	0.63%	-0.13%	-0.13%	-0.39%	-1.58%			-3.35%	-11.88%
0.4					-0.13%		-1.58%				
0.5	1.11%	1.11%	0.63%	-0.13%	-0.13%	-0.39%	-1.58%	-1.58%	-3.35%	-3.35%	-11.88%
0.6							-1.58%				
0.7							-1.58%				
0.8							-1.58%				
1							-1.58%				-11.89%
1.5							-1.20%				

0.5x0.5 cm² (Y=0.5 cm, X=0.5 cm)

ESSx \ ESSy	0	0.2	0.3	0.4	0.5	0.6	0.7	0.8	0.9	1	1.5
0					0.59%		-0.88%				
0.1					0.59%		-0.88%				
0.2					0.59%		-0.88%				
0.3	2.00%	2.00%	1.55%	0.42%	0.42%	0.17%	-1.07%			-2.95%	-12.13%
0.4					0.04%		-1.44%				
0.5	1.56%	1.56%	1.13%	0.04%	0.04%	-0.20%	-1.44%	-1.44%	-3.30%	-3.31%	-12.45%
0.6							-1.56%				
0.7							-2.11%				
0.8							-2.11%				
1							-2.92%				-13.75%
1.5							-7.60%				

Figure S1 – *Profile in the X direction for a 0.5x0.5 cm² field of 6X, acquired with microDiamond (in water) and with film (in solid water).*

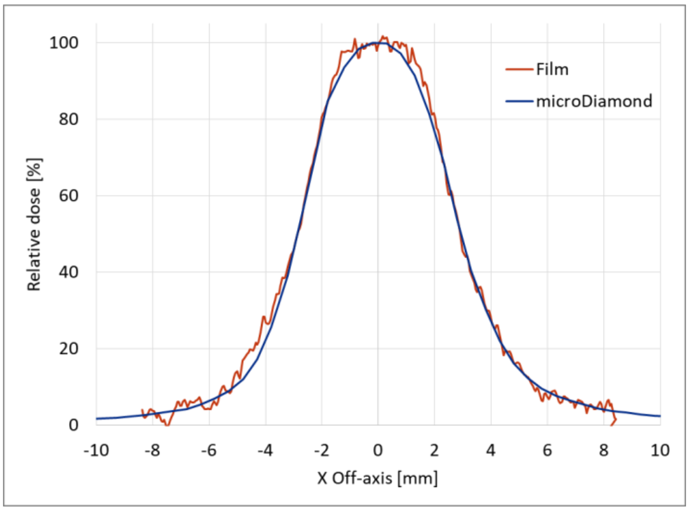

Figure S2 – *Difference between calculated dosimetric (FWHM) and nominal field size, as a function of the ESS parameter, X direction on the left, Y direction on the right, for 0.5x0.5, 1x1 and 2x2 cm*^{2} *fields from 6X.*

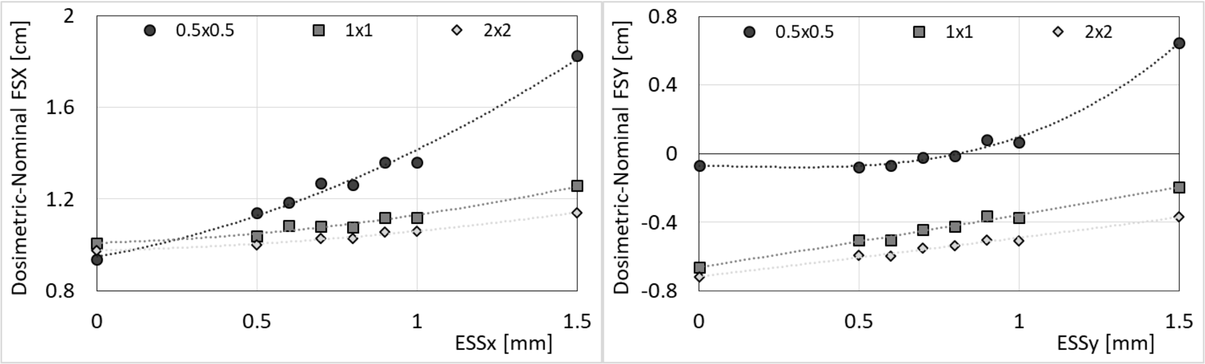

Supplement: Supplementary file 1 — Supporting Information [file ACM2-26-e70315-s001.pdf]
